# Misunderstood, Minimised, Misrepresented: Autistic Young Adults' Experiences of Epistemic Injustices in Healthcare Interactions Around Autism

**DOI:** 10.1111/1467-9566.70124

**Published:** 2025-11-29

**Authors:** Aino‐Mari Uisma, Tuija Virkki, Minna Ylilahti

**Affiliations:** ^1^ University of Jyväskylä Jyväskylä Finland

## Abstract

This article explores autistic young adults' experiences of epistemic injustice in healthcare interactions where autism is invoked and a subject of misaligned sensemakings. We take a particular focus on hermeneutical injustice, which refers to the difficulty of understanding and communicating one's experiences due to gaps and biases in sensemaking resources. Earlier research concerning autistic individuals from this perspective is sparse and mostly theoretical. Our study addresses this gap by conducting abductive thematic analysis on interview and questionnaire data from Finnish autistic young adults. This approach enabled us to recognise biases and hierarchies, such as neuronormativity and epistemic hierarchies, while also describing a new, paradoxical kind of hermeneutical injustice not covered by earlier research. Our results indicate how the often‐intertwined forms of epistemic injustices are related to a lack of representativeness in how autism is understood as well as to how the exclusion of certain kinds of knowledge and knowers, along with the devaluations of expressive styles, impact autistic people's epistemic agency, identities and access to care. We argue for more inclusive epistemic approaches in healthcare that appreciate autistic contributions, recognise the diversity of autistic experience and expression, and challenge the reductive assumptions embedded in diagnostic and professional practices.

## 1 Introduction

Autism is a contested and multifaceted concept. It is traditionally defined in diagnostic manuals as a neuropsychiatric disorder marked by deficits in social, communicative and behavioural skills (WHO 2022; APA 2013). However, this approach—often called the medical model of autism—is challenged by expanding perspectives, such as the neurodiversity paradigm, which frames autism as a natural and neutral variation of human diversity with various presentations and experiences (Anderson–Chavarria 2022, 1334–7). The medical model is increasingly criticised as overly pathologising and not reflecting the diverse lived experiences of autistic^1^ individuals (Catala 2025, 276–9; Anderson–Chavarria 2022, 1327–9).

Amid these tensions, autism is a way to interpret experiences, which in philosopher Fricker’s (2007) terms would be called a hermeneutical tool. Like other diagnoses, it describes, classifies and designates roles and treatment (Jutel 2024, 55). It can also be an embodied way of being (De Jaegher 2013) and both a personal and social identity, shaping how individuals understand themselves and connect with autistic communities (Corden et al. 2021; Cooper et al. 2017). This multifaceted nature illustrates how autism, like many other diagnoses, has escaped the clinical context and gained new social meanings (Jutel 2024; Brinkmann 2016).

Healthcare is a site where these varied and conflicting understandings of autism become visible. Research on healthcare experiences shows how autistic individuals face understandings different from their own: narrow, stereotypical and deficit‐focused understandings of autism rife with gendered and age‐related assumptions (Adams et al. 2025; Cage et al. 2024; De Broize et al. 2022; Milner et al. 2019; Bargiela et al. 2016). These misalignments are described as contributing to negative experiences and barriers to care. Although these studies demonstrate misalignments and tensions, earlier research has not explored them from the perspective of biases and hierarchies in knowledge production and use.

In this article, we use abductive thematic analysis to address this gap by exploring Finnish autistic young adults' experiences of misaligned understandings of autism in healthcare interactions.^2^ These interactions, where autism is invoked and a subject of conflicting interpretations, are situated in various healthcare services, from general and mental healthcare to diagnostic processes. Our focus on young adults aged 18–34 is informed by the changes in multiple areas of life occurring during this period, such as in education, employment and social relationships (Arnett 2007). During this time the role of services can be pronounced for autistic individuals (Drmic et al. 2018, 254–7), making their healthcare experiences important to study. Our data include individuals who have recently visited primary and specialised healthcare and services for autistic individuals, which are often for children and young adults under the age of 29, both in Finland and elsewhere (Duodecim 2024; Howlin 2021).

Theoretically, our article is grounded in critical autism studies' focus on systemic issues within institutions (Woods et al. 2018) and informed by medical sociology's interest in the impact of professional–patient relationships on healthcare experiences (Oh Nelson 2021). We connect these micro‐level healthcare experiences and concerns of critical autism studies for macro‐level oppressive structures and hierarchies by employing the perspective of epistemic injustice (see also Mladenov and Dimitrova 2023), with an emphasis on hermeneutical injustice as the most relevant form of epistemic injustice in the context of our analysis. Epistemic injustice refers to wrongs done to one's capacity as a knower, a capacity ‘essential to human value’ (Fricker 2007, 1, 44). It encompasses various forms of epistemic harm related to knowledge practices, including testimonial injustice, when someone is considered less credible due to stereotypical assumptions, and hermeneutical injustice, which arises when understanding and articulation of experiences are hindered by gaps and biases in shared interpretative resources. The overall approach of epistemic injustice offers tools to explore how biases and hierarchies in sensemaking are embedded in broader social structures and how these injustices are often systemic and intertwined with other injustices (Fricker 2007, 1–8, 27–9).

We approach healthcare interactions around autism as informed by intersecting hierarchies of healthcare and neuronormativity. In healthcare interactions, patients are routinely positioned as less capable knowers than professionals (Carel and Kidd 2017), and a growing body of research explores epistemic injustices in healthcare, particularly as experienced by marginalised groups (for review, see Jonas et al. 2025). Differences in communication styles between autistic and nonautistic people create mutual misunderstandings (Milton 2012), further complicating healthcare interactions between patients and professionals (Shaw et al. 2024). Drawing on the recent theoretical work of Catala et al. (2021), we acknowledge how neuronormativity—the hegemonic assumptions, norms and practices that place certain social, communicative and cognitive styles as better than others—creates additional hierarchies where autistic people are considered deviant or disordered in their diverging from these neuronormative ideals. We understand autistic people's healthcare experiences as influenced by this dual position of autistic patienthood.

In this article, we ask what kinds of epistemic injustices autistic young adults encounter in healthcare interactions around autism. In doing so our study sheds light on epistemic injustices at the heart of autistic people's poor healthcare experiences, opening avenues for further research and addressing these injustices. As previous research on specifically hermeneutical injustices experienced by autistic people has been mostly theoretical and research into healthcare experiences of autistic people has not adequately addressed the social dimensions of knowledge, our empirical study is an important contribution to both areas of research.

### 1.1 Epistemic Injustice

In her seminal theoretical work, Fricker (2007) describes two kinds of epistemic injustices: testimonial and hermeneutical. Testimonial injustice occurs when prejudice leads someone to unjustly deflate the credibility of someone else because of stereotypical assumptions tied to their identity. For example, autistic people may not be believed due to a perceived lack of credibility associated with autism (Williams and Jobe 2025). Hermeneutical injustice occurs when gaps in collective interpretative resources make certain social experiences difficult to understand and communicate to others. This unintelligibility stems from certain groups being excluded from knowledge production due to structural identity prejudices (Fricker 2007, 147–55). Since its emergence, scientific and popular knowledge concerning autism has been produced mostly without the perspectives of autistic people, as they have often been deemed less capable authors of their own experience (Pellicano and Den Houting 2022, 384–6). This has resulted in a lack of representativeness and the circulation of ideas about autism criticised as misrepresentative and harmful (Catala 2025, 268–9; Yergeau and Huebner 2017). As we are interested in tensions stemming from gaps and biases in sensemaking resources, our study takes a pronounced focus on the two main kinds of hermeneutical injustice.

Conceptual hermeneutical injustice (Fricker 2007, 155–6) concerns the lack of shared representative sensemaking resources, such as concepts and labels, to describe an experience. For example, autistic women may not be recognised as autistic due to a lack of knowledge about their experiences and existence (Bargiela et al. 2016). Hermeneutical injustice can also be expressive, occurring when someone is not understood or believed because of how they express themselves (Fricker 2007, 160–1). For instance, even though stimming—repetitive movements or vocalisations—may be important for autistic individuals to self‐soothe and communicate emotions and thoughts, others may not understand it as meaningful (Kapp et al. 2019).

The primary harm of hermeneutical injustice is being prevented from making sense of experiences that are in one's interest to have understood (Fricker 2007, 162). For autistic people, understanding and having others understand autism is important (Botha et al. 2022). Additionally, hermeneutical injustices can contribute to a loss of confidence in one's own epistemic abilities, limitations in using and gaining knowledge, and harm to one's identity in how they are perceived by others and themselves (Fricker 2007, 161–68). For example, autistic people may be treated with assumptions grounded in diagnostic descriptions (Nicolaidis et al. 2015) or internalise negative perceptions about themselves (Leedham et al. 2020, 138–9).

### 1.2 Hermeneutical Injustice and Autism

We adopt an expanded view of hermeneutical injustice by drawing on Amandine Catala's theoretical work, which often discusses autism. Her work emphasises the structural dimensions of hermeneutical injustice by highlighting complex and interconnected relationships between different kinds of epistemic injustices, epistemic authority—whether someone is perceived as a credible knower—and epistemic agency (Catala 2023, 2025, 251–2). Importantly, we employ a clarified, structural understanding of epistemic agency as the capacity to express, use or produce knowledge, shaped by interaction between individual capabilities and structural conditions such as stereotypes and norms (Catala 2025, 35–45). This approach is useful for recognising neuronormativity and epistemic hierarchies of healthcare as background conditions potentially influencing healthcare interactions.

Investigating neuronormativity is important in healthcare contexts: Diagnostic descriptions construe autism in terms of disorders and deficits (WHO 2022; APA 2013), a language which exemplifies neuronormativity (Catala 2023, 247). Deficits in medical conceptualisations of autism may negatively impact autistic individuals' agencies in social spheres (Anderson–Chavarria 2022), such as healthcare interactions. Guided by Catala’s (2025, 172–80; Catala et al. 2021) work, we recognise neuronormativity as a key background condition that marginalises autistic ways of being by contributing to conceptual gaps, privileging normative expressive styles and setting representational limits on hermeneutical tools such as the concept of autism that can profoundly influence self‐understanding and identity.

Additional important background conditions in our analysis are the epistemic hierarchies in healthcare, which often privilege formal medical training and theory over patient knowledge (Carel and Kidd 2017). Medical practice may objectify the individual, facilitating care while also risking dehumanisation through restricted agency and neglect of the individual and social context (Timmermans and Almeling 2009, 22–3). Likewise, preference for quantitative measurement in current diagnostics may obscure relational and contextual dimensions (Brinkmann 2016, 75–88).

We investigate this hierarchy of knowledges from the perspective of Catala’s (2025, 35–45) pluralist epistemology. Moving beyond narrow, propositional definitions, we recognise experiential knowledge—tacit, embodied and affective forms of knowing—and their marginalised position in the knowledge hierarchies of healthcare. Earlier research reports a lack of understanding or acknowledgement of the lived experiences and perspectives of autistic patients (Adams et al. 2025; Cage et al. 2024; Markham 2019). Given the embrace of patient agency and involvement in patient‐centred care (Oh Nelson 2021), this tension underscores the importance of exploring how hierarchies of knowledge come into play in healthcare experiences.

## 2 Research Context and Methodology

### 2.1 Data

Our broader dataset consists of 24 interviews and 107 questionnaire responses from Finnish autistic adults, collected in early 2024. Invitations to participate were shared by national and local autism organisations and, with permission, in online groups. Some participants were also recruited by earlier participants. In this article, we focus on a substudy of young adults aged 18 to 34, comprising 23 interviews and 44 questionnaire responses. We conducted interviews and questionnaires to include participants with different preferences and modes of communication for a wider range of perspectives and experiences. The interviews and questionnaires focused on shared themes and core questions concerning personal meanings and experiences of autism, well‐being and healthcare and service experiences. Although our analysis covers both kinds of data, when illustrating our results, we employ quotes mainly from our interviews, as they tend to be more descriptive due to the flexible, semi‐structured nature of our interviews.

Ethical sensitivity is required when researching autistic individuals and their healthcare experiences. We followed the guidelines by the Finnish National Board on Research Integrity (TENK, 2019) and good research practices of autism research (Hobson et al. 2023). We adhered to the principles of informed consent by providing information about the research and participant rights, easy‐read documents and opportunities for discussion with the researchers before, during and after participation.

Furthermore, we took several steps to avoid causing our participants distress exceeding their everyday experiences. Participants could choose between a semi‐structured interview (in‐person, video call or phone) and an online questionnaire according to their preferences. We shared a written overview of the interview process and sample questions with interviewees beforehand. The topics discussed were familiar to our participants, as they related to their everyday experiences, and they could discuss them to the extent they were comfortable with. We consulted a data protection officer to make sure we complied with data protection regulations and paid specific attention to data processing and anonymisation to protect our participants. To improve readability, we refer to our participants using pseudonyms when presenting our results. Our study does not feature research designs, such as deviation from informed consent, that would require an ethical review according to TENK (2019) guidelines and the guidelines by the ethics committee of the University of Jyväskylä.

Our data encompass various experiences with healthcare services in Finland. Most are related to neuropsychiatric diagnostic processes and are situated within public healthcare, although our data also feature some experiences from private healthcare. Diagnostic processes may vary depending on age, location and current services used. Generally, they are conducted in multidisciplinary teams using tools such as questionnaires, observations and interviews. Although many other countries use the latest diagnostic manuals, Finland uses ICD‐10, with a shift to ICD‐11 expected in 2026 (Duodecim 2024). Diagnostic trends (Hinkka–Yli–Salomäki et al. 2014) reflect broader international patterns of rising diagnosis rates and a male‐skewed gender ratio (Zeidan et al. 2022).

Many of our participants use their diagnosis to access support services, including those for individuals with neuropsychiatric diagnoses. These are often designed for children and adults under 29 and require a formal diagnosis (Duodecim 2024). Our participants most frequently use psychosocial rehabilitative programmes intended to support them in everyday life, study life and work life. According to national guidelines (Duodecim 2024), support should be planned and implemented with autistic individuals and their significant others and be informed by their individual situations and goals. In addition to autism‐specific services, our data cover experiences within primary and specialised healthcare more broadly.

Our participants have diverse support needs and demographic and social backgrounds. The majority of them are women (62%) and cisgender (78%). Most (64%) are aged 26 to 34 and live in urban areas (79%). The most common highest education level is secondary school (54%), with noticeable portions of those with comprehensive education (10%) or higher education (31%) as their highest level of education. They are mainly students (31%) or employed (28%), with notable numbers of unemployed and pensioners (12% each). These are disability pension for individuals under the age of retirement with long‐term decreased ability to work due to illness or disability. They may be temporary, with the goal of returning to work or study, or paid indefinitely (Kela 2025).

We recruited adults who identify with autism, and consequently, our data represent various positions towards formal diagnosis. In accordance with Ardeleanu et al. (2024), we recognise the value of including undiagnosed autistic individuals for a more nuanced understanding of autistic lives and barriers to formal diagnosis. Around half of our participants (55%) hold a formal diagnosis, whereas others are seeking, were denied or chose not to seek diagnosis due to concerns over, for example, stigmatisation. Some have recently navigated diagnostic processes; others are in early childhood.

### 2.2 Analysis

To analyse the data, we applied the main principles of abductive analysis (Timmermans and Tavory 2012) to thematic analysis (Braun and Clarke 2022). This form of abductive thematic analysis, involving an iterative and reflexive movement between data and theory, enabled us to remain open to the possibilities of new empirical findings and refine our theoretical understanding throughout the analysis process (Khurshid et al. 2025). We first familiarised ourselves with the data through re‐reading and note‐taking while enhancing our theoretical sensitivity by exploring multiple theoretical frameworks related to autism. This established an iterative process of revisiting data from various perspectives. Initially, we coded the entire dataset based on our tentative research interest in how autism is understood. Our attention was then drawn to the tensions and misalignments between how our participants and others understood autism, particularly in healthcare interactions.

To explore this topic further, our revisiting of the data focused on the healthcare context. Alongside autism research, we engaged with studies in medical sociology concerning interactions between professionals and patients. This provided theoretical lenses for re‐coding data and generating themes differentiating various types of tensions and misalignments. The theoretical sensitivity offered by critical autism studies helped us identify tensions between a view of autism as a neutral form of human diversity and a pathological, deficit‐focused view (the theme of social vs. medical models). Medical sociology, with its theorisation of hierarchies between professionals and patients, helped us identify other tensions, for example, between professional expertise and lay knowledge (the theme of epistemic agency).

Next, we defamiliarised ourselves from these initial thematisations by asking questions on issues that remained puzzling. Why and how do such tensions and misalignments become pronounced in healthcare contexts, which one might assume to offer autistic individuals the support and understanding they need? How could we gain insight into the multidimensional processes and structural mechanisms that give rise to these tensions and misalignments? The theme of epistemic agency, formed during an earlier round of analysis, crystallised essential dimensions of these questions. This insight led us to the multifaceted theoretical framework of epistemic injustice.

We next began to reframe the phenomenon and generate new insights by exploring theoretical perspectives from the broader framework of epistemic injustice. We tested their explanatory power in analysing the processes and mechanisms behind tensions and misalignments. During the ‘alternative casing’ phase, we experimented with applying general and healthcare‐specific theorisations of epistemic injustice to our data alongside those developed within critical autism studies.

Through this recursive process of theorisation, we identified the most compelling explanatory frameworks, particularly those focused on hermeneutical injustice and the concept of neuronormativity. These frameworks enabled us to re‐code our data in dialogue with theory, using revised codes to generate themes. For instance, the code ‘masking is not understood’ was re‐coded as an instance of conceptual hermeneutical injustice. This iterative engagement enabled us to uncover the underlying mechanisms and power relations that (re)produce various types of tensions and misalignments in healthcare interactions around autism.

Importantly, while refining our analysis, we encountered theoretical misfits because earlier research on expressive hermeneutical injustice did not fully reflect the nuances we observed. This surprise, central to abductive analysis (Timmermans and Tavory 2012), prompted further theorisation. The abductive approach enabled us to notice this gap and, through continued analysis, flesh out this observation, refine existing theory and contribute novel insights to the conceptual landscape.

## 3 Three Problems of Epistemic Injustices

Based on the abductive thematic analysis of our data, we generated three themes highlighting the dimensions of epistemic injustices in healthcare interactions around autism. Our first theme, ‘Autistic Identities and the Problem of Narrow Understandings of Autism’, focuses on the tensions created by conflicting understandings of autism and their impact on service access and autistic identities. The second theme, ‘Experiential Knowledge and the Problem of Denied Epistemic Authority’, explores the epistemic hierarchies in healthcare and the devaluation of experiential knowledge and autistic people's credibility as knowers. The final theme, ‘Autistic Masking and the Problem of Appearing “Too Normal”’, discusses hermeneutical gaps around masking and the devaluations of expressive styles. These themes are interconnected in complex and nuanced ways, and together, they contribute to a more multifaceted understanding of how epistemic injustices manifest in problematic healthcare interactions around autism, along with the contextual conditions and adverse implications linked to such injustices.

### 3.1 Autistic Identities and the Problem of Narrow Understandings of Autism

Many of our participants view autism as more than just a medical diagnosis. They see it as an essential and inseparable part of their identity. As Noa writes: ‘Autistic traits are not something that accompany me, like depression in the life of a depressed person, but rather autism is the same thing as me’. Autism is also often perceived as a neutral or positive form of human diversity, encompassing diverse experiences and presentations. These perspectives contribute to a richer and more nuanced understanding of autism.

However, some of our participants have encountered different understandings of autism in healthcare interactions. These include deficit‐focused language and approaches implying one is, as described by Ruska, ‘somehow defective or deficient’. Such understandings of autism, aligned with the medical model of autism, are seen as too focused on outdated stereotypes and lacking acknowledgement of what autistic people themselves consider important.

For example, Havu finds diagnostic questionnaires ‘somehow restricted, they are looking for only one dimension of autism and not considering the various other related aspects’. Our participants point out these stereotypes refer more often to boys and men, and there is a lack of knowledge about ‘how autism manifests in women's lives’, as Sara notes. According to Catala (2025, 278), the medical sphere (re)produces gender biases and fails to represent the diversity of autistic presentations—both structurally in the diagnostic tools and interactionally during diagnostic interviews, where these tools are used to interpret interviewees' behaviours and responses.

Based on our analysis of participants' shared negative experiences, we contend the understanding of autism within healthcare tends to be hermeneutically nonrepresentative (Catala 2025, 269–70) as it fails to reflect how autistic people make sense of and experience autism. Autistic people may also find it difficult to use their own alternative, more representative sensemakings. For example, Miska attempted to convey his experience of ‘special interests’—intense and focused interests that are a common characteristic in autism and part of the diagnostic criteria (WHO 2022)—during the diagnostic interview:

I remember when they asked, for example, about my interests, and then I showed my plushie, saying that I have about 20 of these. And then the interviewer asked if I display them somewhere. And when I said no, … I got the impression like it somehow undermined it, that if I collect something, they should be on display. I shouldn’t have the plushies stored here and take them out when I feel like it. … Somehow, they should be like some model trains, arranged on shelves in numerical order.

As his experience did not line up with the idea of specific kinds of ‘special interests’, he felt dismissed and judged as not having ‘special interests’ at all. This speaks to how alternative sensemakings, like this more nuanced understanding of ‘special interests’, may not be appreciated.

The problems of the medical model, which simultaneously fails to represent autistic individuals and excludes alternative sensemakings, are evident in many interactions described by our participants. In the context of diagnostic processes, our participants discuss how they have found it difficult to be recognised as autistic if they deviate from the narrow scope of the medical model. Heidi shares that during her diagnostic process, ‘I felt there was a lot of questioning, especially at the beginning of the examinations, about the possibility of my autism because I have studied at university and moved into working life’. Her educational and professional background was considered incongruent with what is expected from autistic people, leading to scrutiny of her eligibility to continue in the process. Instead of encountering consideration for the individual experiences and meanings of autism, one may find themselves mechanistically evaluated against rigid medical criteria.

Similarly, Sara describes her experience of seeking to enter the diagnostic process: ‘But unfortunately, doctors use the ICD disease classification in a curt way, ticking off criteria like this one is not met, neither is this one, that one is met, that one is too weak’. This evaluation against a narrow understanding of autism exemplifies a key problem with nonrepresentative conceptualisations of autism: Autistic individuals risk having their autism questioned or denied whenever they do not appropriately align with the medical model. Consequently, individuals with various experiences may be rendered unrecognisable when judged against this narrow understanding of autism. These constitute classical examples of conceptual hermeneutical injustice, where gaps and biases in how autism is understood contribute to difficulty in being understood.

As illustrated in the experiences quoted above, autistic people have trouble being recognised as autistic and may also be denied access to diagnostic processes and the services that require formal diagnosis. This lack of representativeness constitutes a practical challenge, potentially hindering access to care—an especially pressing concern for autistic young adults, who may have a pronounced need for support (Drmic et al. 2018, 254–7). Additionally, autistic individuals may pre‐emptively limit what they share for fear of dismissal; Maria gave up on seeking a diagnosis ‘because I had just heard from others that autistic traits are not well‐known, especially in women’. The nonrepresentative understanding of autism may further entrench itself through exclusion of alternative experiences.

Beyond these barriers to care, these quotations illustrate how the problem of nonrepresentativeness is intimately related to identity. Because autism is important for many of our participants, dismissal of their identities can be hurtful. Maria felt that her therapist did not fully believe she was autistic, leaving her feeling that ‘you don't see me wholly as the way I myself would like to be seen’. Not receiving affirmation from healthcare professionals is described as causing ‘imposter syndrome’ and doubt over the validity of their identities and experiences.

Consequently, autistic individuals may be hindered from identifying as autistic. Sara shares how she tried to make the importance of the diagnostic process intelligible to a nurse, explaining that ‘this affects my quality of life so much and how I could identify and reflect on myself, that I could become a good person’. This illustrates how impactful identification as autistic can be and the problem of being prevented from doing so. In Catala’s (2025, 262) terms, this would be an instance of existential hermeneutical injustice, where one is prevented from becoming ‘who they are’ due to their identities being judged and denied based on nonrepresentative understandings of autism.

Autistic people may suffer further injuries to their identities when the nonrepresentative understanding of autism is applied to them. Our participants share how they have been treated with assumptions they find false, such as their having sensory issues or no social skills. These assumptions may be occasional or more characteristic of the interactions. For example, Nelli describes her rehabilitative service where she constantly faced assumptions that made her feel that ‘I am not seen there as a person and as myself but rather as some kind of “bingo card” of traits’.

This exemplifies another injury inflicted upon autistic identities: They may become perceived and treated in a misrepresentative manner that misconstrues their identities. One may come to ‘count as’ (Fricker 2007, 165–6) the narrow, stereotypical kind of autism they do not consider representative. Application of diagnostic knowledge and categories this way appears to be problematic since it risks obscuring and misconstruing individual presentations and situations. Diagnosis is a classification that generalises and summarises multiple cases to organise illness in a meaningful way, but it does not capture individual presentation (Jutel 2024, 14–15). Consequently, care may be guided by these assumptions, such as being treated as less capable. Julia shares how ‘very often it has been questioned why I am applying to a university of applied sciences’ based on her diagnoses. This made her feel that ‘it gives a very deflated feeling, like I am not capable of anything’.

This illuminates the harm of having one's identity misconstrued. When autistic individuals are faced with these nonrepresentative understandings of autism and are treated accordingly, it may influence their self‐perceptions. Mira writes how the diagnostic process has impacted her self‐perception: ‘Through the diagnostic process, I have come to see as problematic even those traits that I did not previously consider problematic (e.g., paying attention to details)’. This too illustrates an identity harm (Fricker 2007, 165–6), where one internalises a nonrepresentative view of themselves. Given the relationship between how one perceives autism and their psychological well‐being (Cooper et al. 2023; Cooper et al. 2017), this kind of internalisation of negative views is concerning.

### 3.2 Experiential Knowledge and the Problem of Denied Epistemic Authority

Our participants describe healthcare interactions where their perspectives and sensemakings are not adequately acknowledged. They may not be consulted to begin with, like Olivia, who shares how in her diagnostic interview ‘so many questions focused on external coping instead of personal experience’. When one does share their own experiences and perspectives, they may be easily disregarded. Sara shares how in her attempt to seek access to the diagnostic process, she was told that ‘but you are studying and working, so you are coping’ even though she herself felt that ‘I'm not coping with my head’. Similarly, Nelli shares how within her diagnostic process her experience was actively dismissed:

I remember one thing that seems to be difficult for everyone to understand. I don’t get how it’s so … difficult. For example, when we were talking about executive functioning and how I can’t physically do something—I may stand by the sink and scream internally at myself to wash the dishes, but it just doesn’t happen. My hands just don’t obey. And the psychiatrist said—we were talking about medicine—that everyone would like to get motivation from a pill bottle. And I was like, this is not about motivation, it’s about my executive functioning not working.

These participants employ diverse knowledge concerning tacit, embodied and affective ways of knowing we may call experiential knowledge (Catala 2023, 259) to make sense of their experiences, but contributions featuring such knowledge are not always appreciated. This constitutes an example of conceptual hermeneutical injustice, where reluctance to acknowledge sensemakings that utilise experiential knowledge hinders one from being understood.

Such a tendency to favour professional knowledge and perspectives is a commonly described feature of epistemic hierarchies of healthcare (Carel and Kidd 2014, 2017). As experiential knowledge is not deemed important enough to consider, we can see how it is treated as a less authoritative kind of knowledge. This devaluation of experiential knowledge is indicative of a wider problem: Autistic people may be perceived as less credible producers, holders and users of knowledge.

Within this hierarchy, autistic people and their knowledge may even be subjugated: Miska shares how he felt his claim of being annoyed as a child whenever dinosaurs were called ‘dinos’ was tested by practitioners who would ‘intentionally’ call them ‘dinos’ during an interview within his diagnostic process. He saw that ‘it felt a bit like testing, and then I felt like I failed the test. They tried to dig out some reaction, and when it didn't come, they concluded that “okay”’. Instead of treating his contribution as knowledge worthy of acknowledgement, it was reduced into something to be tested and evaluated by the more authoritative professionals. This exemplifies how hermeneutical injustice may work to undermine epistemic authority. When a person's knowledge and contributions are perceived as less important or intelligible, so are they themselves situated as less authoritative knowers.

Autistic individuals' epistemic authorities may be further undermined by how autism is understood. Our participants share instances where their contributions were questioned or devalued based on their autistic identity. Sointu writes how she was told by a doctor that ‘you have somatic pain because of your autism. You are not really sick’. This worrying dismissal of her sickness based on her autism indicates that the label of autism may carry connotations of poor epistemic authority and lead to testimonial injustice. These injuries to epistemic authority may also result in being treated differently after one discloses their autism. Our participants share instances of being spoken to like a child, and, as Tuuli describes, after disclosing autism, ‘you'd be seen as somehow dim‐witted and like … [the physician] had to explain everything and speak with simplified language’, suggesting that autistic individuals are perceived as less capable. Although meant to improve access to care and understanding, the label may conversely herald assumptions and discredit them with concerning implications.

Together, these prejudiced views of autistic individuals and epistemic hierarchies of healthcare may engender a denial of authority and contribute to difficulty in being understood. Our participants report their needs going unrecognised when they were limited in how they could participate in the discussion. Heidi was told by her psychiatrist ‘before I had even said anything’ that ‘it'll be really difficult for you to get [rehabilitation] and it may not even make sense to apply since you are working [in education]’. Without considering Heidi's perspective, the psychiatrist risks denying her access to care based on how her situation appears initially to the professional, not her perspective.

Furthermore, the care received may be ill‐informed. If experiential knowledge is disregarded, insight into socially situated experiences and lives may be lost. Some of our participants discuss how their care should acknowledge how living as an autistic person in a society that does not take kindly to autistic modes of being has impacted them. However, this need may not be understood by healthcare professionals, like in Olivia's case: ‘I tried to share that I feel like I have to cover up autism all the time, and it's exhausting and stressful and that stress creates these obsessive thoughts’ but ‘then the nurse was like … well why can't you just be yourself? … Feels like they didn't really understand the minority trauma that comes with growing up autistic in this society’. For her, understanding her life and health in the context of a neuronormative society was important, but her nurse did not appreciate this relationship. This illustrates how lack of acknowledgement of experiential knowledge can obscure wider socially situated experiences and their influence on one's health and needs.

Beyond barriers to appropriate care, autistic individuals may be further impacted by being denied authority. Our participants share how disregard for their contributions and their credibility can be deeply disconcerting. Tuuli describes how she felt after her physician spoke to her differently after noticing her diagnosis: ‘I think it's really scary because the information is available for those professionals … you should get expert care. And just, what if I'm not believed or heard?’ This worry over whether they will be treated as credible knowers of their own lives and experiences can be pervasive.

Furthermore, for some of our participants, experiences of being treated as less credible knowers are common and contribute to the sense their perspective does not matter. Julia describes how it may be interpreted ‘that I'm in a good situation, so to speak, even though I don’t feel that way myself, but it probably looks good on the surface’ and that ‘I feel a bit like it doesn't even matter’. This demonstrates how denial of epistemic authority can foster a sense of alienation and disillusionment with healthcare.

### 3.3 Autistic Masking and the Problem of Appearing ‘Too Normal’

Many of our participants have adapted their behaviour to appear less autistic and, as Sumu describes, ‘spent a lot of time in my life to learn things that make me appear “more normal”’. This suppression of autistic traits and projection of more socially acceptable ones, often to avoid victimisation, is called autistic masking (e.g., Radulski 2022). Academic explorations of masking are recent, following Bargiela et al. (2016), but the phenomenon has been discussed by autistic people for longer (see Gerland 1996).

As a relatively new and community‐created concept, masking is not always well‐known: Our participants discuss how some healthcare practitioners lack understanding and knowledge of masking. Tuuli recalls a psychiatrist telling her that her being autistic ‘is not possible because I look into the camera’ during a remote session, prompting her to reflect that it ‘feels like many don't even know about masking’ and that ‘autists have the ability to act and do things differently from what they might feel is best for them’.

This lack of knowledge and understanding of masking in healthcare constitutes a classical and problematic example of conceptual hermeneutical injustice (Fricker 2007). Autistic people may have the label for masking and make sense of their experiences through it, but some healthcare professionals lack awareness and understanding of it. This gap is problematic because it may contribute to misrecognition or denial of one's autism. As Salla writes about her experience of the diagnostic process: ‘If I mask “too well” I'm not taken seriously because masking is not understood enough’.

Although lack of understanding is attributed mostly to healthcare, the hermeneutical injustice of masking is more nuanced. Some participants reflect on how, despite having the label to describe the experience, their understanding of masking as a complex, elusive phenomenon is impartial. As Miska says: ‘Even I don't understand how much it influences what I do’. Some also reflect on how their lack of awareness and possibilities to address masking has impacted their healthcare experiences negatively.

Beyond these conceptual gaps, masking relates to expressive dimensions of knowledge. As these quotations illustrate, when one masks ‘too well’, they may be questioned or not taken seriously. This indicates a curious phenomenon where appearing ‘too normal’ may be a problem and lead to discrediting based on how one presents themselves, a kind of expressive hermeneutical injustice (Fricker 2007). Our participants frequently discuss how their ‘too normal’ expression styles—including speaking ‘too well’, nonverbal communication such as eye contact, and dressing ‘too normal’—engendered questioning and dismissing their autism or the experienced difficulties they attempt to communicate. Nelli quotes how a psychiatrist commented on her communication style and questioned her request for referral to diagnostic services:

Well, I think it’s very nice to interact with you. I mean, you’re not like a profoundly autistic person who doesn’t talk or … You express yourself very well, are you really sure that you are autistic or that you want this diagnosis?

Expressive styles deemed ‘too normal’ appear to be judged incongruent with expectations concerning autistic expression. These expectations of, for example, poor verbal skills and lack of eye contact are aligned with the medical model of autism and its focus on assumed deficits in social communication, as discussed in our first theme. Our participants reflect on how these expectations are too narrow to describe the range of expressive styles autistic people have. Maria discusses how the expectation that autistic people always have ‘slowness of speech, learning’ is quite ‘outdated’. Similar to Chapman and Carel (2022), here we see how different forms of epistemic injustice influence each other. Because of nonrepresentativeness, those who do not appear sufficiently deficient in social communication may be questioned or dismissed. This shows how expressive hermeneutical injustices are informed by conceptual hermeneutical injustices, namely, the lack of representativeness in how autism is understood.

This problem reveals a flaw in the tendency to privilege professional knowledge and interpretation over patient knowledge. Lacking concepts to understand the diversity of autistic expressions and how masking may influence them, healthcare professionals are prone to misinterpreting appearances they see as ‘too normal’. As discussed in the second theme, the tendency to privilege professional knowledge and interpretation over autistic people's contributions is troubling because autism is not easily perceived visibly. Appreciation of experiential knowledge could help reconcile this problem. As Miska says, it might be better ‘not to look at the outwardly visible behaviour’, instead hoping that ‘what's inside me would have as much weight as how it appears on the outside’.

Furthermore, masking may be encouraged by the structures of healthcare interactions. Reflecting on masking, Aurora states that ‘I realised that maybe I have to stop pretending like I'm coping so well, because clearly I'm not and I need this help’, but ‘there wasn't really enough time for me to be genuinely myself in those situations’. This indicates how, besides hermeneutical gaps and hierarchies, environmental aspects require consideration in how they contribute to this multifaceted and complex problem.

Furthermore, we must ask whether non‐normative expression styles would be better received. Although appearing ‘too normal’ may engender misunderstandings and devaluation, so can non‐normative expression styles. For example, Aurora shares how her expressions of extreme pain were interpreted as her being ‘such a cheerful person who just laughs’, and Nelli's expressive ways of movement were assumed to be ‘a problem with motor skills’ to be corrected.

Indeed, autistic people may find themselves in a difficult position where paradoxically both non‐normative and normative communication styles may result in misinterpretation or devaluation. This is an insidious kind of expressive hermeneutical injustice, where either way one may face discrediting. This paradoxical position is best illustrated by Hilla, who writes how, because of masking,

at the same time people don’t believe my difficulties and yet they get angry and misunderstand if I don’t mask. So, it’s contradictory. They say be yourself, and then it’s misinterpreted when I am.

## 4 Discussion and Conclusion

Drawing from qualitative data concerning experienced problems in healthcare interactions around autism, our study explored various kinds of epistemic injustices autistic young adults encounter in healthcare. Our analysis suggests that epistemic injustices manifest as a lack of representativeness in how autism is understood, limitations on what kinds of sensemaking resources are usable and devaluation of expressive styles. These epistemic injustices are often interconnected, hinder our participants from being understood and appear to be informed by epistemic hierarchies of healthcare and deficit‐focused understandings of autism. Importantly, our analysis illuminates how epistemic injustices may contribute to practical challenges, such as difficulty accessing appropriate care, and impact identities of autistic individuals. Our analysis also discussed how these injustices work to undermine the epistemic authority and agency of our participants, effectively situating them as less credible knowers of their own experiences and identities and limiting their participation in knowledge production.

These problematic experiences are crucial to address because the period of young adulthood current to our participants is important for identity‐making and the subsequent life phases (Arnett 2007). The importance of support services during young adulthood can be seen in the hopes our participants place in healthcare to address their needs and the despair they feel when help is not received (Drmic et al. 2018, 254–7). Their experiences are located within the wider landscape of the increasing problematisation of traditional medical understandings and pluralisation of meaning‐making around autism. As these struggles and tensions are reflected in the negative experiences shared by our participants, they provide insight into how such conflicts can play out in healthcare interactions around autism.

This empirical investigation produces a more nuanced understanding of expressive hermeneutical injustices encountered by autistic individuals, expanding beyond earlier research focused on non‐normative expressive styles (Catala et al. 2021). Our finding that appearing ‘too normal’ may likewise engender dismissal echoes research on denial of identity (Botha et al. 2022, 437), difficulty accessing care (Bargiela et al. 2016) and the complex relationships between stigma, masking and identity (Perry et al. 2022). However, these findings have not been discussed as instances of expressive hermeneutical injustice. Conducting our analysis abductively helped us observe this theoretical gap and describe a paradoxical kind of expressive hermeneutical injustice. The finding that both non‐normative and normative expression styles may engender discrediting and dismissal brings new perspectives to theories of epistemic injustice.

Expressive hermeneutical injustice is, importantly, related to the contradictory workings of neuronormativity. Neuronormativity contributes to the unintelligibility of non‐normative expressive styles by privileging normative styles while upholding stereotypical and deficit‐focused understandings of autistic communication (Catala et al. 2021). In line with Chapman and Carel's (2022) research on interlocking epistemic injustices, this paradox manifests in our analysis as the simultaneous devaluation and misunderstanding of non‐normative expressive styles and ‘too normal’ expressive styles. These are, in turn, compounded by a lack of knowledge about masking and a narrow understanding of autistic communication. Fuelled by neuronormativity, this paradox places autistic individuals in a difficult position, where misunderstanding and devaluation may be suffered no matter how one expresses themselves.

Furthermore, neuronormativity contributes to discrediting autistic people and their contributions and upholding nonrepresentative conceptualisations of autism. Our results converge with research on experienced stereotypical assumptions (Adams et al. 2025; Nicolaidis et al. 2015) and difficulties in being recognised and understood as autistic (De Broize et al. 2022; Milner et al. 2019; Bargiela et al. 2016). In line with earlier research (Adams et al. 2025; Cage et al. 2024; Markham 2019), our study describes a limited appreciation of experiential knowledge and the patient's perspective. This reflects how epistemic hierarchies of healthcare often privilege professional knowledge and perspectives (Carel and Kidd 2017). These insights underscore the importance of acknowledging neuronormativity and epistemic hierarchies of healthcare as important background conditions that may inform epistemic injustices.

These injustices could be addressed by acknowledging the multiplicity of experiences and sensemakings around autism. As Chapman and Carel (2022) highlight, a shift from narrow understandings of autism may be achieved by critically examining deficit‐focused approaches and supporting alternative, nonpathologised understandings of autism. To reconcile the paradoxical expressive hermeneutical injustice described in this study, a good place to start would be re‐evaluating assumptions about autistic communication and improving understanding of masking. Furthermore, our study highlights the importance of addressing the diagnostic practices and structures of healthcare institutions. Diagnoses are generalisations and summaries of multiple cases (Jutel 2024, 14–15). Their rigid application may obscure and misrepresent individual presentations and needs.

Clinical practice also requires epistemic humility (Medina 2013): Healthcare professionals should reflect on the limitations of their knowledge and embrace patient contributions. We concur with Catala (2023, 253–61) that genuine epistemic authority on autism should recognise how social position influences understanding and that propositional knowledge should be informed by experiential, critical and diverse insights of autistic people. This would be a promising step towards healthcare encounters that are better informed by individual needs and more closely aligned with the ideals of patient‐centred care (Oh Nelson 2021).

## Author Contributions

**Aino‐Mari Uisma:** conceptualization, data curation, investigation, methodology, formal analysis, funding acquisition, writing – original draft, writing – review and editing. **Tuija Virkki:** conceptualization, formal analysis, methodology, supervision, writing – original draft, writing – review and editing. **Minna Ylilahti:** conceptualization, formal analysis, methodology, supervision, writing – original draft, writing – review and editing.

## Funding

The lead author is employed as a doctoral researcher at the University of Jyväskylä and, prior to this position, received a grant from the Finnish Society for Disability Research for data collection.

## Ethics Statement

The Human Sciences Ethics Committee of the University of Jyväskylä does not require ethical review for studies of this kind. Our study follows the ethical guidelines of the Finnish National Board on Research Integrity (TENK) and the principles of informed consent.

## Consent

Information about the study, data privacy and participant rights was provided in advance and before participation in writing and, for interviewees, orally. All written information and consent forms were available as easy‐read versions. The participants had the opportunity to discuss and ask questions regarding their participation and rights prior to, during and after participation. All participants provided written or oral informed consent, which was recorded prior to participation.

## Conflicts of Interest

The authors declare no conflicts of interest.

## Permission to Reproduce Material From Other Sources

The authors have nothing to report.

## Data Availability

Data are not available as we did not ask participant consent to share data publicly. We acknowledge that our data cover sensitive experiences and that not making data available would support participant trust and comfort. Metadata: https://doi.org/10.17011/jyx/dataset/102790.

## Endnotes

^1^
We recognise the various positions on appropriate terminology and ways of speaking about autism. We aligned our choices with what most (though not all) of our participants prefer and with the findings of numerous studies (e.g., Dindar et al. 2023), which highlight the preference, among autistic individuals, for identity‐first and nondeficit‐based language, such as *autistic* or *autist*.

^2^
Despite our focus on experienced misalignments, we do not imply that all healthcare interactions shared by our participants were experienced as problematic. Our aim is not to discredit the value of care or to generalise about healthcare and professionals with diverse positions and practices. Rather, we explore how healthcare interactions are experienced and identify problematic patterns. Examining positive experiences is an important matter for further research, but it is beyond the scope of this study.
